# Use of next generation sequence to investigate potential novel macrolide resistance mechanisms in a population of *Moraxella catarrhalis* isolates

**DOI:** 10.1038/srep35711

**Published:** 2016-10-24

**Authors:** Ya-Li Liu, Dong-Fang Li, He-Ping Xu, Meng Xiao, Jing-Wei Cheng, Li Zhang, Zhi-Peng Xu, Xin-Xin Chen, Ge Zhang, Timothy Kudinha, Fanrong Kong, Yan-Ping Gong, Xin-Ying Wang, Yin-Xin Zhang, Hong-Long Wu, Ying-Chun Xu

**Affiliations:** 1 Department of Clinical Laboratory, Peking Union Medical College Hospital, Peking Union Medical College, Chinese Academy of Medical Sciences, Beijing 100736, China; 2 Wuhan National Laboratory for Optoelectronics, Huazhong University of Science and Technology, Wuhan, Hubei 430074, China; 3 Binhai Genomics Institute, BGI-Tianjin, BGI-Shenzhen, Tianjin 300308, China; 4 Tianjin Translational Genomics Center, BGI-Tianjin, BGI-Shenzhen, Tianjin 300308, China; 5 Department of Clinical Laboratory, the First Affiliated Hospital of Xiamen University, Xiamen, China; 6 Charles Sturt University, Leeds Parade, Orange, New South Wales 2687, Australia; 7 Centre for Infectious Diseases and Microbiology Laboratory Services, ICPMR – Pathology West, Westmead Hospital, University of Sydney, Darcy Road, Westmead, New South Wales 2145, Australia

## Abstract

Although previous studies have confirmed that 23S rRNA gene mutation could be responsible for most of macrolide resistance in *M. catarrhalis*, a recent study suggested otherwise. Next generation sequence based comparative genomics has revolutionized the mining of potential novel drug resistant mechanisms. In this study, two pairs of resistant and susceptible *M. catarrhalis* isolates with different multilocus sequence types, were investigated for potential differential genes or informative single nucleotide polymorphisms (SNPs). The identified genes and SNPs were evaluated in 188 clinical isolates. From initially 12 selected differential genes and 12 informative SNPs, 10 differential genes (*mboIA*, *mcbC*, *mcbI*, *mboIB*, *MCR_1794*, *MCR_1795*, *lgt2B/C*, *dpnI*, *mcbB*, and *mcbA*) and 6 SNPs (C619T of *rumA*, T140C of *rplF*, G643A of *MCR_0020*, T270G of *MCR_1465*, C1348A of *copB*, and G238A of *rrmA*) were identified as possibly linked to macrolide resistance in *M. catarrhalis*. Most of the identified differential genes and SNPs are related to methylation of ribosomal RNA (rRNA) or DNA, especially *MCR_0020* and *rrmA.* Further studies are needed to determine the function and/or evolution process, of the identified genes or SNPs, to establish whether some novel or combined mechanisms are truly involved in *M. catarrhalis* macrolide resistance mechanism.

*M. catarrhalis* is a prominent pathogen that causes acute otitis media in children and lower respiratory tract infections in adults (such as exacerbations of chronic obstructive pulmonary disease)^1^,^2^, resulting in significant socioeconomic burden on healthcare systems globally. Because of the high prevalence of β-lactamase-producing *M. catarrhalis* isolates, macrolide initially appeared to provide a safe alternative to β-lactam antibiotics for the treatment of respiratory illnesses with a low risk of serious side effects, especially for children^3^. Although most *M. catarrhalis* isolates are still susceptible to macrolide in many countries^4^,^5^,^6^, with a minimal inhibitory concentration (MIC)_90_ of 0.25 g/L, macrolide-resistant *M. catarrhalis* have been reported in several studies worldwide^7^,^8^,^9^. In 2012, we reported for the first time that ribosomal mutation was crucial for creating macrolide-non-susceptible *M. catarrhalis*, and that A2330T (position referring to *M. catarrhalis* 23S rRNA gene, GenBank number NR_103214.1) mutation was related to high-level macrolide-resistance^10^. In the following three years, several studies confirmed that indeed A2330T mutation of the 23S rRNA gene could lead to isolates with high-level macrolide resistance (MIC > 256 g/L)^11^,^12^,^13^,^14^.

Findings from a recent study in Japan revealed that *M. catarrhalis* strains with high level macrolide resistance also exhibit mutations in ribosomal proteins L4 (V27A and R161C) and L22 (K68T)^14^. Interestingly and in contrast to *M. catarrhalis*, mutation of the 23S rRNA gene is usually not the main reason for macrolide-resistance in other bacterial species (such as *Streptococcus pneumoniae, Streptococcus pyogenes*, etc^15^,^16^,^17^). Moreover, multilocus sequence typing (MLST) results from our previous study showed a very high level of heterogeneity among *M. catarrhalis* isolates^10^,^13^. Given the above background, it is possible that several mechanisms are involved in *M. catarrhalis* macrolide resistance.

In order to investigate potential novel mechanisms involved in macrolide resistance by *M. catarrhalis*, with special emphasis on new relevant genes or informative single nucleotide polymorphisms (SNPs), we studied in detail 2 macrolide resistant and 2 susceptible *M. catarrhalis* isolates using genomic sequencing. The aim was to screen for other possible resistance genes or mutations (apart from 23S rRNA gene mutation) responsible for macrolide resistance in *M. catarrhalis*, and to further confirm the findings in a large collection of clinical isolates.

Firstly, we intended to gain further insights into whether A2330T mutation is solely responsible for macrolide resistance in *M. catarrhalis*, or whether other mechanisms, including methylase, efflux pump, or other genes or mutations^14^, alone or in combination, are involved. Secondly, given that macrolide resistant *M. catarrhalis* are so different from other macrolide resistant cocci, we assumed that macrolide resistance in *M. catarrhalis* may be associated with the distinct genomic background of this organism.

In order to answer the two questions above, comparative genomics and multiple molecular typing methods for genetic population were used in this study. Based on the genome-wide data of two pairs of macrolide susceptible and resistant isolates (n = 4), and further evaluation in 188 clinical isolates, we found that six informative SNPs and ten differently expressed genes, possibly contribute to macrolide-resistance in *M. catarrhalis*.

## Materials and Methods

### Statement

All the authors confirm that all experiments were performed in accordance with relevant guidelines and regulations. Study materials involved in our study are clinical isolates, no human subjects (including the use of tissue samples) were included in the present study, so informed consent was not required in this study which has been approved by the Human Research Ethics Committee of Peking Union Medical College Hospital (No. S-424).

### Bacterial isolates

Genome sequencing was performed on two pairs of *M. catarrhalis* isolates (2 susceptible and 2 resistant) arbitrarily chosen. The details of the two susceptible isolates are as follows:

1. Strain 13R13726 was *M. catarrhalis* isolated in 2013 from a purulent sputum of a 33 year old outpatient woman with lower respiratory tract infection. The erythromycin and azithromycin MIC of this organism was 0.125 g/L each.
2. Strain 11XR1696 was an *M. catarrhalis* isolate with erythromycin and azithromycin MICs of 0.125 g/L each, and was isolated in 2011 from a 40-year-old woman admitted in hospital with lower respiratory tract infection.

Details of the two resistant isolates are as follows:

1. Strain 13R13685 was *M. catarrhalis* with erythromycin and azithromycin MICs of >256 g/L each. This organism was isolated in 2013 from the purulent sputum of a 61-year-old outpatient man with chronic obstructive pulmonary disease.
2. Isolate 11XR4410 was *M. catarrhalis* with erythromycin and azithromycin MICs of >256 g/L each, and was isolated from the purulent sputum of a 62-year-old inpatient with lower respiratory tract infection.

In addition, 21 macrolide-resistant *M. catarrhalis* (including 11XR4410 and 13R13685) and 167 macrolide-susceptible *M. catarrhalis* (including 11XR1696 and 13R13726) isolates from our previous study^13^, were randomly selected to evaluate the comparative genomic results (Table 1).

### Antimicrobial susceptibility testing

As previously published, all the isolates (n = 188) were tested for susceptibility to erythromycin and azithromycin using the disc diffusion (Thermo Fisher, Oxoid, Basingstoke, UK) method according to CLSI 2010 guideline^18^. And the macrolide-resistant *M. catarrhalis* isolates were confirmed by E-test (bioMérieux, Marcy l’Etoile, France) method to get the minimum inhibitory concentrations (MICs). *Staphylococcus aureus* ATCC 25923 was used for quality control.

### DNA extraction

Isolates were grown overnight at 35 °C on blood agar plates and DNA extracted using the QIAamp DNA Mini Kit (Qiagen, Dusseldorf, Germany) following the manufacturer’s instructions.

### Molecular typing for genetic population study

Multilocus sequence typing (MLST) (http://mlst.warwick.ac.uk/mlst/dbs/Mcatarrhalis), which was performed on the 2 pairs of macrolide-susceptible and resistant *M. catarrhalis* isolates, was inferred from the best hit homologs of *abcZ* (ATP-binding protein), *adk* (adenylate kinase), *efp* (elongation factor P), *fumC* (fumarate hydratase), *glyRS* (glycyl-tRNA synthetase beta subunit), *mutY* (adenine glycosylase), *ppa* (pyrophosphate phospho-hydrolase), and *trpE* (anthranilate synthase component I) genes present in each genome, in accordance with the *M. catarrhalis* MLST scheme developed previously (Table 2) 19.

Pulsed-field gel electrophoresis (PFGE) and *copB* polymerase chain reaction–restriction fragment length polymorphisms were performed on the four isolates as previously described^10^.

### Next-generation genomic sequencing (NGS)

Genome sequencing was performed on the two pairs of *M. catarrhalis* isolates (one pair susceptible and the other resistant). DNA libraries were constructed with genomic DNA using kits provided by Illumina Inc. according to the manufacturer’s instructions. Libraries with an insert size of 500-bp were prepared for each isolate. Methods for DNA manipulation, including formation of single-molecule arrays, cluster growth and paired-end sequencing, were performed on an Illumina Hiseq 2500 sequencer according to standard protocols. The Illumina base-calling pipeline was used to process the raw fluorescent images and call sequences. Raw reads of low quality from paired-end sequencing (those with consecutive bases covered by fewer than five reads) were discarded. The bioproject accession number for the four isolates (11XR4410, 11XR1696, 13R13726 and 13R13685) is PRJNA338378.

### Differential gene definition in macrolide resistant and susceptible groups

The paired-end reads from each of the four genome sequenced isolates were mapped to a previously published *M. catarrhalis* reference genome, the BBH18 reference genome (GenBank accession number: CP002005.1) and *M. catarrhalis* isolate E22 plasmid pLQ510 (GenBank accession number: NC_011131.1) using Burrows-Wheeler Alignment (BWA) software. Nucleotide base coverage of each gene from each of the isolates on the BBH18 and plasmid pLQ510 genomes was assessed using Samtools mpileup packages (http://samtools.sourceforge.net/). Based on the Samtools mpileup results, the average coverage for each gene was calculated. If the gene coverage was different between the resistant and susceptible groups by either being present or absent, or if present, by significantly (P < 0.05) different levels of gene coverage, those genes were considered as differential genes.

For SNPs, the number of genome nucleotide bases of a test isolate that were similar to those of the reference genome (ref) were determined. Likewise the number of test isolate genome nucleotide bases that were different (alt) to the reference genome were also determined for each isolate. High quality SNPs were defined as SNPs that satisfied the following criteria: alt/(alt+ref) >0.95 (which means to be different from the reference genome nucleotide base) or alt/(alt+ref) ≤0.05 (which means to be similar to the reference genome nucleotide base). A high quality SNP which satisfied the criterion alt/(alt+ref) >0.95, and appeared in at least one isolate, was considered to be an informative candidate SNP.

### PCR screening of differential genes

**Figure 1:**
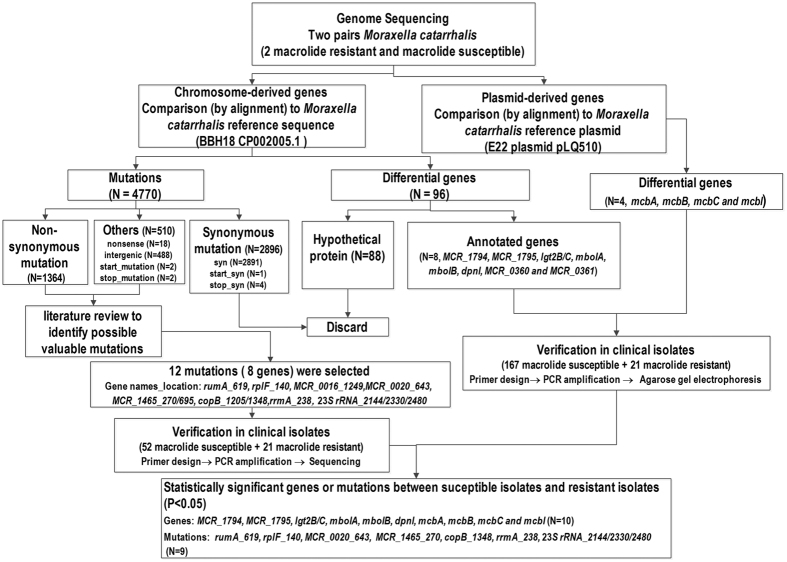
Flow chart of the study.

PCR was performed on 188 *M. catarrhalis* isolates (21 macrolide resistant, and 167 macrolide susceptible) derived from Peking Union Medical College Hospital (PUMCH): 2010–2013, to detect the following identified differential genes as per definition above; *mboIA*, *mcbC*, *mcbI*, *mboIB*, *MCR_1794*, *MCR_1795*, *lgt2B/C*, *dpnI*, *mcbB*, *MCR_0360*, *MCR_0361*, and *mcbA* (see Table 3 for primer sequences and Supplementary Table S1 for full description of the genes). A detailed flow chart of the study is shown in Fig. 1. A standard PCR protocol was used for all PCRs. The PCR protocol for *mboIA*, *mcbC*, *mcbI*, *mboIB* genes used a standard PCR protocol comprising 94 °C for 5 min, followed by 30 cycles of 94 °C for 30 sec, 55 °C for 30 sec, 72 °C for 40 sec, and a final extension step of 72 °C for 7 min. For the *MCR_1794*, *MCR_1795*, *lgt2B/C*, *dpnI*, *mcbB* genes, a standard PCR protocol comprising an extension time of 1 min at 72 °C was used. Finally, for *MCR_0360*, *MCR_0361*, and *mcbA* genes, a standard PCR protocol comprising an extension time of 1 min 30 sec at 72 °C was used (Fig. 1).

### PCR and sequencing analysis of the informative candidate SNPs

PCR was performed to detect *rumA*, *rpIF*, *MCR_0016*, *MCR_0020*, *MCR_1465*, *copB* and *rrmA* genes (see Table 3 for primer sequences) among 73 *M. catarrhalis* isolates (all 21 macrolide resistant isolates available and 52 randomly selected macrolide susceptible isolates, from PUMCH). A standard PCR protocol was used for all PCRs. The PCR protocol for *rumA*, *MCR_0016*, *MCR_0020*, *MCR_1465*, *copB* and *rrmA* genes used an initial annealing temperature of 94 °C for 5 min, followed by 35 cycles of 94 °C for 30 sec, 55 °C for 30 sec, 72 °C for 90 sec, and a final extension step of 72 °C for 7 min. For the *rpIF* gene, a standard PCR protocol comprising 94 °C for 5 min, followed by 35 cycles of 94 °C for 30 sec, 50 °C for 30 sec, 72 °C for 90 sec, and an extension time of 7 min at 72 °C, was used. We further investigated the 23S rRNA gene sequence changes in a 934-bp region in all the 73 *M. catarrhalis* isolates as per our previous study^10^. Due to limited budget, only 73 isolates instead of the 188 were tested for the presence of the identified informative candidate SNPs (Fig. 1).

DNA sequencing was performed on the 73 isolates using the same primers used for PCR amplification, providing bidirectional coverage. The obtained sequences were aligned to those of the wild type GenBank reference *M. catarrhalis* strain, BBH18; GenBank accession number. NR_103214.1).

## Results

### General genome features of the studied *M. catarrhalis* isolates

Detailed descriptions of the isolates used in this study are shown in Table 1. The isolates studied represent a clinically diverse collection of *M. catarrhalis* isolates from sputum of patients treated at PUMCH. The genome sizes of the four *M. catarrhalis* isolates that were studied in detail (2 susceptible and 2 resistant) ranged from 1.85 to 1.96 Mbp, with a mean size of 1.91 Mbp (Table 1). BBH18 is the previously published reference *M. catarrhalis* genome^20^.

### MLST and PFGE of *M. catarrhalis* genome isolates

MLST analyses of the 2 pairs of susceptible and resistant *M. catarrhalis* isolates studied in detail, showed that each of the four genomes represented a different sequence type (ST), including NP-ST-4 (NP-ST: Denotes sequence types not present in the MLST database at the time of analysis), NP-ST-5 (NP-ST: Denotes sequence types not present in the MLST database at the time of analysis), ST312, and ST327. Furthermore, 2 novel sequence variants for the *abcZ* (*abcZ* 61) and *efp* (*efp 34*) alleles were present in strains 13R13726 and 11XR1696, respectively (Table 2). However, isolate 13R13685 was broadly similar to 11XR4410, albeit with two exceptions (*abcZ and ppa*). All the four isolates belonged to *copB* II. We utilized PFGE analysis to determine the clonal relationship of the 4 isolates, and four pulsotypes were found (data not shown), suggesting origination from different clones.

### Comparative genomics differential genes

The paired-end reads from each of the 4 isolates studied in greater detail (13R13726, 13R13685, 11XR1696 and 11XR4410) were mapped to the BBH18 reference genome (GenBank accession number: CP002005.1) and *M. catarrhalis* isolate E22 plasmid pLQ510 (GenBank accession number: NC_011131.1) using BWA. When compared to the BBH18 reference genome, 96 differential genes were detected, including 88 hypothetical protein genes and 8 annotated genes (*mboIA*, *mboIB*, *MCR_1794*, *MCR_1795*, *lgt2B/C*, *dpnI*, *MCR_0360*, and *MCR_0361*). For the *M. catarrhalis* E22 plasmid pLQ510 comparison, 4 differential genes (*mcbA*, *mcbB*, *mcbC*, and *mcbI*) belonging to annotated genes, were detected (Fig. 1).

### Comparative genomics informative SNPs

The paired-end reads from each isolate (13R13726, 13R13685, 11XR1696 and 11XR4410) were mapped to the BBH18 reference genome (GenBank accession number: CP002005.1) using BWA. SNPs occurring in at least one isolate were considered. A total of 19489 SNPs were detected when each of the four isolates was compared to BBH18 reference genome, and 4770 SNPs were found to be unique to susceptible (13R13726 and 11XR1696) or resistant isolates (11XR4410 and 13R13685). Considering that different non-synonymous mutations may cause the same alternation of amino acid, all the 4770 SNPs analyzed in this study were confirmed after assessment for both synonymous and non-synonymous mutations. In *M. catarrhalis*, there are four identical copies of rRNA (16S, 23S, and 5S rRNA genes) operons in which the 16S and 23S rRNA genes are interspersed with genes encoding tRNAs for isoleucine and alanine^14^. Among the 4770 SNPs observed to be unique to either susceptible or resistant isolates, 29 were from the rRNA gene, including A2144T, A2330T, and C2480T mutation of 23S rRNA, which were mainly contributed by the resistant isolates (Table 4) as previously noted^7^. Moreover, among the two macrolide-resistant *M. catarrhalis* isolates (11XR4410 and 13R13685), A2330T and C2480T mutations could be detected in all the four different operons of these isolates, while A2144T mutation was only detected in four operons of strain 11XR4410.

### Distribution of the identified differential genes and informative candidate SNPs in clinical isolates

Because of the diverse genetic background of the 4 isolates studied in greater detail, not all of the identified differential genes and informative candidate SNPs were considered to be highly related to macrolide resistance without further confirmation in a large collection of clinical isolates. By reviewing relevant literature (Supplementary Table S1), 12 of the identified annotated genes (*mboIA*, *mcbC*, *mcbI*, *mboIB*, *MCR_1794*, *MCR_1795*, *lgt2B/C*, *dpnI*, *mcbB*, *MCR_0360*, *MCR_0361*, and *mcbA*) and 12 informative candidate SNPs (A2144T, A2330T, and C2480T of 23S rRNA, C619T of *rumA*, T140C of *rplF*, A1249G of *MCR_0016*, G643A of *MCR_0020*, T270G and G 695A of *MCR_1465*, A1205C and C1348A of *copB*, and G238A of *rrmA*) were considered relevant for macrolide resistance. The 12 selected annotated genes and 12 candidate SNPs were further tested in 188 *M. catarrhalis* isolates of clinical origin (21 macrolide resistant and 167 macrolide susceptible) and 73 isolates (21 macrolide resistant *M. catarrhalis* isolates and 52 macrolide susceptible *M. catarrhalis* isolates), respectively (Fig. 1).

**Figure 2:**
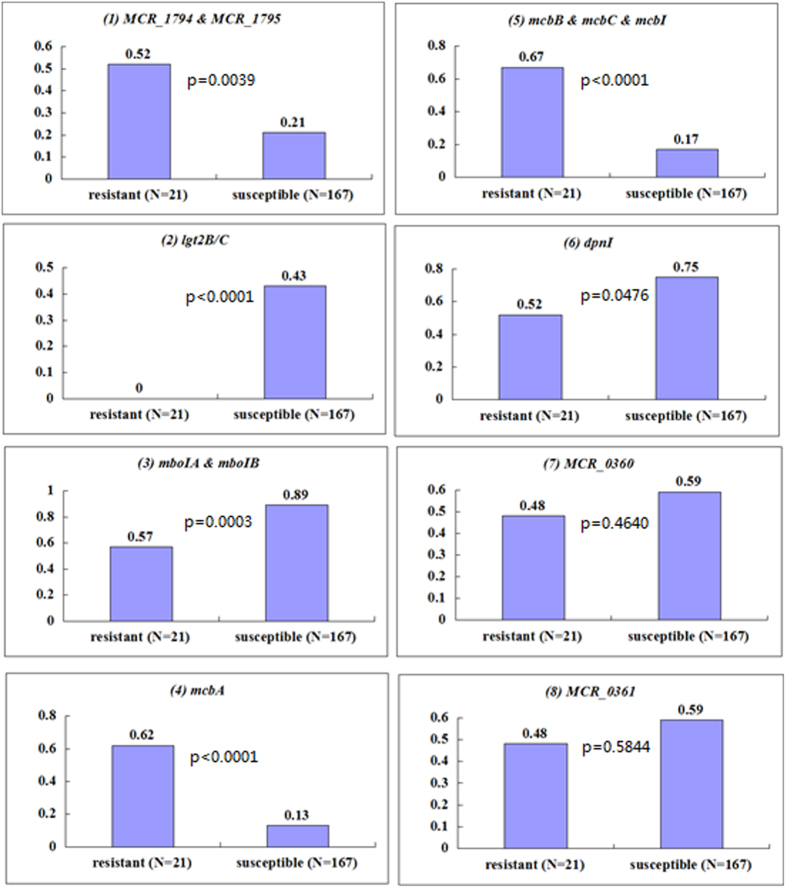
Prevalence of 12 annotated genes (including 10 differential genes) amongst 21 macrolide resistant and 167 macrolide susceptible *M. catarrhalis* clinical isolates. Axis of ordinates: percentages of gene-positive isolates/total isolates (%). Charts 1, 3, and 5 above, the prevalence rates are for 2 or 3 genes combined.

The frequency distribution of the 12 annotated genes between the macrolide resistant (n = 21) and susceptible (n = 167) clinical isolates was compared using a χ2-test. The presence of *mboIA* (p = 0.0003), *mcbC* (p < 0.0001), *mcbI* (p < 0.0001), *mboIB* (p = 0.0003), *MCR_1794* (p = 0.0039), *MCR_1795* (p = 0.0039), *lgt2B/C* (p < 0.0001), *dpnI* (p = 0.0476), *mcbB* (p < 0.0001), and *mcbA* (p < 0.0001) genes was statistically different between the two groups, while the presence of *MCR_0360* (p = 0.4640) and *MCR_0361* (p = 0.5844) was very similar in the two groups (Fig. 2).

**Figure 3:**
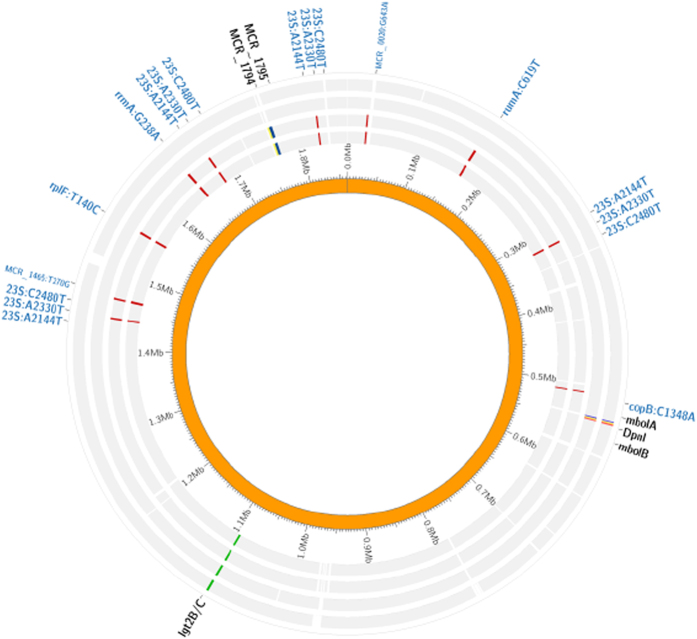
Diagrammatic representation of the distribution of the 9 SNPs and 6 genes in the genomes of the four *M. catarrhalis* isolates. From the inside to the outside, the circles represent the sequences of strains BBH18 (reference genome for *M. catarrhalis*), 11R4410, 13R13685, 13R13726 and 11XR1696. The gene *lgt2B/C* exists both in the resistant and susceptible isolates but with different levels of coverage (resistant isolates 0.376/susceptible isolates 1).

**Figure 4:**
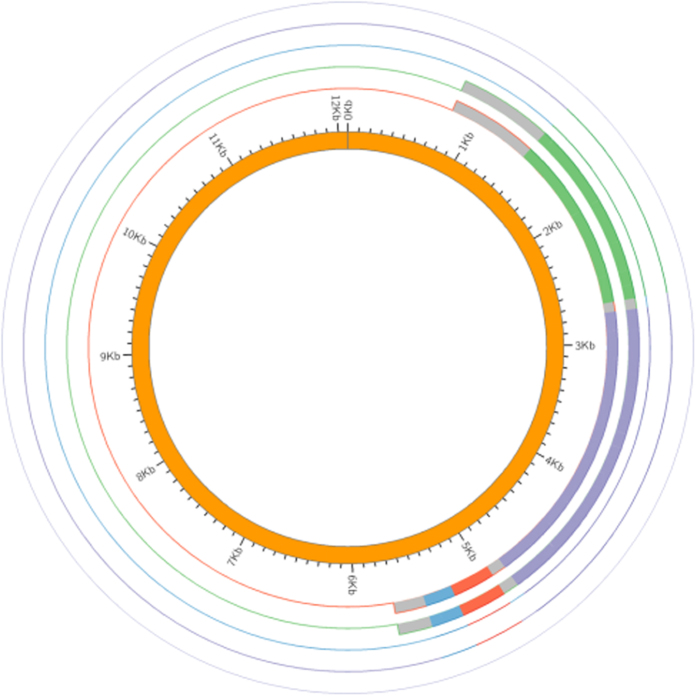
Diagrammatic representation of the *mcbA*, *mcbB*, *mcbC*, *mcbI* genes in the plasmids of the four *M. catarrhalis* isolates. From the inside to the outside, the circles represent the sequences of plasmid pLQ510 (one of the two currently characterized *M. catarrhalis* plasmids), 11R4410, 13R13685, 13R13726 and 11XR1696. The colors green, purple, red and blue represent plasmid derived *mcbA*, *mcbB*, *mcbC* and *mcbI* genes, respectively.

Comparisons of the frequency distribution of informative candidate SNPs in the two groups (resistant, n = 21 vs. susceptible, n = 52) using a χ2-test, indicated that the presence of the A2144T, A2330T, and C2480T mutation of 23S rRNA, C619T of *rumA*, T140C of *rplF*, G643A of *MCR_0020*, T270G of *MCR_1465*, C1348A of *copB*, and G238A of *rrmA*, was statistically different between the two groups (p < 0.05). In contrast, the presence of A1249G mutation of *MCR_0016*, G695A of *MCR_1465*, and A1205C of *copB* genes, was very similar in the two groups (p > 0.05) (Table 5). Diagrammatic representations of the positions of the 9 SNPs and 10 genes as confirmed in the genomes of the four *M. catarrhalis* isolates are shown in Figs 3 and 4.

## Discussion

Based on our literature review, we found that very few studies have examined the molecular mechanisms involved in *M. catarrhalis* macrolide resistance. In most studies, 23S rRNA gene mutation is singled out as being responsible for the majority of cases of macrolide resistance^10^,^11^. However, whether 23S rRNA gene mutation is the only mechanism leading to macrolide resistance in *M. catarrhalis* remains unknown, though the presence of macrolide-resistant strains without any 23S rRNA gene mutations seem to suggest otherwise.

Comparative genomics is a practical tool which has been widely used in the study of drug resistance mechanisms^21^. Specifically, if the isolates to be compared are derived from the same patient and have similar genetic background, comparative genomics can provide some important information, including discovery of some novel mechanisms. Unfortunately, in our study, we couldn’t find sufficient numbers of *M. catarrhalis* isolates with the same MLST or pulsed field gel electrophoresis types, as there was considerable genetic diversity among the isolates. As such, it is clear that not all the SNPs and genes identified in this study can be considered to be highly associated with macrolide resistance, hence had to confirm some of them in a large collection of clinical isolates.

**Figure 5:**
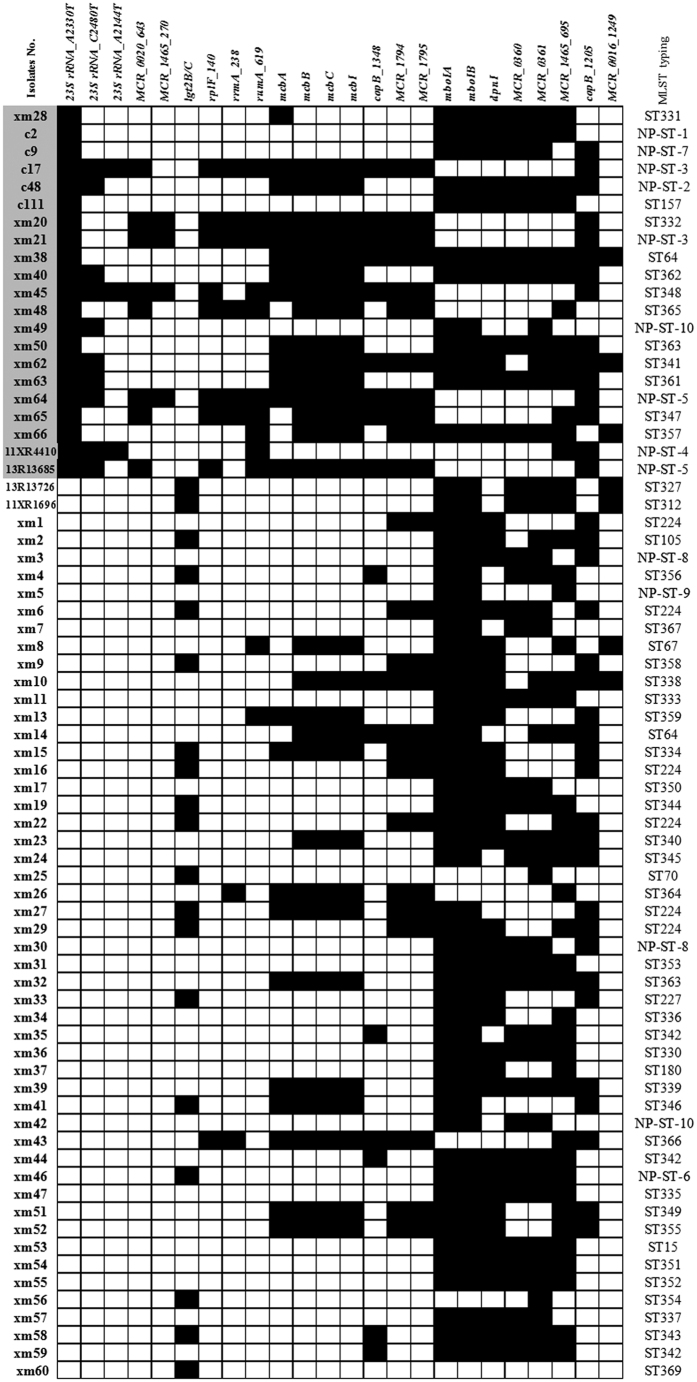
Distribution of 12 selected annotated and differential genes and 12 selected informative SNPs in macrolide susceptible (n = 52) and resistant (n = 21) *M. catarrhalis* clinical isolates. The top 21 rows with gray background on the isolate ID (extreme left) are the 21 macrolide resistant isolates, while the rest represent the susceptible group (n = 52; randomly selected from 167 isolates). Black cells: denotes presence of genes or SNPs; blank cells: absence of genes or SNPs. NP-ST: denotes sequence types not present in the MLST database at the time of analysis. The NP-ST-1 ~ NP-ST-10 listed the 10 new MLST sequence types but not presented the MLST database. The two or more strains with the same ST but different genes/SNPs profiles are listed below: ST 224 (6 strains): xm1, xm6, xm16, xm22, xm27, and xm29; ST342 (3 strains): xm35, xm44 and xm59; ST64: xm14 and xm38; NP-ST-3: c17 and xm21; NP-ST-5: xm64 and 13R13685; NP-ST-8: xm3 and xm30; NP-ST-10: xm42 and xm49.

Based on the comparative genomics results combined with evaluation in a large collection of clinical isolates, some genes and SNPs considered possibly involved in macrolide resistance were identified (Figs 1 and 5). Most of the identified genes and SNPS are related to the methylation of ribosomal RNA (rRNA) or DNA, especially *MCR_0020* and *rrmA* (Supplementary Table S1). Due to limited relevant literature on *M. catarrhalis* genome or resistance to macrolide, some of the genes (such as *mboIA* and *mboIB*) (Supplementary Table S1) and informative candidate SNPs (such as C619T of *rumA* and T140C of *rplF*) (Supplementary Table S1) identified in this study have not been previously reported. It is unclear how methylation of rRNA and rDNA is associated with macrolide resistance in *M. catarrhalis.* Thus further investigation, including function of the gene, and crystal structure of the protein involved, and how these relate to macrolide resistance, is needed.

Furthermore, we analyzed the distribution of the identified 12 differential genes and 12 SNPs in the two groups (resistant, n = 21 vs. susceptible, n = 52), in order to find possible gene combinations associated with macrolide resistance (Fig. 5). Among the 12 candidate SNPs, 5 (23S rRNA_A2144T, 23S rRNA_A2330T, 23S rRNA_C2480T, *MCR_0020_*G643A, *MCR_1465_*T270G) were only detected in the resistant group, whilst among the differential genes, only one gene (*lgt2B/C*) was identified in the susceptible group. The remaining SNPs and differential genes were detected in both groups. In addition, among the remaining SNPs, C619T of *rumA*, T140C of *rplF*, and G238A of *rrmA* mutations, were always found together in the resistant group, and no definite pattern was obvious in the susceptible group. Moreover, the distribution frequency of the *mcbB*, *mcbC* and *mcbI* genes was similar between the two groups, and so was that of *mboIA* and *mboIB*, *MCR_1794* and *MCR_1795* genes. These findings suggest that these genes are associated with each other when they function in the cell.

Interestingly, the identified 12 SNPs and 12 differential genes could also be used to differentiate individual isolates even between two isolates which shared the same MLST type, such as strain xm21 (NP-ST-3) and c17 (NP-ST-3).

Based on the above results, we surmise that the molecular mechanism of macrolide resistance might not be as simple as previously thought^10^,^11^,^12^,^13^,^14^, and that some genes or SNPs (such as *MCR_0020* and *rrmA*) might be involved in this process. Many of the identified genes are related to the methylation of ribosomal RNA (rRNA) or DNA, and may solely, or in combination, with one another or with 23S rRNA gene mutation, be responsible for macrolide resistance in *M. catarrhalis.* However, the functions of these identified genes or SNPs, and the crystal structure of their translated proteins, are still unknown. We consider these findings as hypothesis generating and exploratory, requiring confirmation in the future to fully elucidate some of these findings.

This study has several limitations. First, the four isolates used as the main anchor of the study were chosen arbitrarily. It’s possible that a different set of *M. catarrhalis* isolates would have yielded different candidate differential genes or informative SNPs. Second, Samtools pileup pipeline can yield less SNPs than other pipelines, therefore some SNPs potentially related to macrolide resistance might have been missed. Third, the selection of candidate differential genes and informative candidate SNPs was mainly based on reviewing relevant literature and further evaluation in 188 clinical isolates; it is possible that some relevant genes and SNPS may have been overlooked due to limited bioinformatic analysis. Fourth, none of the 10 annotated differential genes were assessed for expression under normal cultural conditions and/or in the presence of macrolides. And finally, it is not possible to rule out that the other 88 hypothetical proteins and 1862 SNPs not mentioned are not involved as they could have hidden some potentially important macrolide resistance genes. More studies are needed to fully understand the mechanism of macrolide resistance by *M. catarrhalis*. Our limited budget was a hindrance to carrying out more detailed studies.

## Additional Information

**How to cite this article**: Liu, Y.-L. *et al*. Use of next generation sequence to investigate potential novel macrolide resistance mechanisms in a population of *Moraxella catarrhalis* isolates. *Sci. Rep.* **6**, 35711; doi: 10.1038/srep35711 (2016).

## Supplementary Material

Supplementary Information

## Figures and Tables

**Table 1:** General genome features of the four *M. catarrhalis* isolates.

Strain	Source country	Isolate source	No. of Contigs	Base No. in Contigs	% GC	Patients’ information			
						Age	Gender	Year*	Diagnosis
BBH18 [20]	Denmark	Blood	1	1,863,286	41.70%	Not available	Not available	Not available	Bloodstream infection
13R13726	China	Sputum	25	1,893,027	41.49%	33	female	2013	Lower respiratory tract infection
13R13685	China	Sputum	26	1,957,031	41.36%	61	male	2013	Chronic obstructive pulmonary disease
11XR1696	China	Sputum	18	1,851,137	41.64%	40	female	2011	Lower respiratory tract infection
11XR4410	China	Sputum	22	1,931,664	41.44%	62	male	2011	Lower respiratory tract infection

^*^Year of isolation.

**Table 2:** MLST results of the four *M. catarrhalis* isolates used in this study.

Strain ID number	*abcZ*	*adk*	*efp*	*fumC*	*glyBeta*	*mutY*	*ppa*	*trpE*	ST	*copB* genotype
13R13726	61^a^	28	3	3	14	36	17	2	327^b^	II
13R13685	8	3	2	2	17	15	8	2	NP-ST-5^b^	II
11XR1696	3	22	34^a^	9	8	3	8	2	312^b^	II
11XR4410	3	3	2	2	17	15	3	2	NP-ST-4^b^	II

^a^New allele.

^b^New ST; NP-ST: denotes sequence types not present in the MLST database at the time of analysis.

**Table 3:** List of primers for PCR screening of macrolide resistant relevant genes and informative SNPs.

Gene name	Primers names and primers sequences (5′-3′)	(Positions on the genome or plasmid sequences)	PCR product size (bp)
*MCR_1794*	t2re-F: ^1764527^AATTCAAGATATTTTTCACGCCCTT^1764551^	CP002005.1	631
	t2re-R: ^1765157^CAGACCATTTACAAAATACAAAAGG^1765133^		
*MCR_1795*	dcm-F: ^1765056^TGTGTATTCTAATTCTATTGGCTGT^1765080^	CP002005.1	548
	dcm-R: ^1765603^TTGCCACTGTTTTTAGTGGTATTGG^1765579^		
*lgt2B/C*	gll-F: ^1089676^TGCCTTTGAGTTTTTTGATGCAGTA^1089700^	CP002005.1	629
	gll-R: ^1090304^ATTTTTTGCCATAATCAGCTAAAGT^1090280^		
*mboIA*	mboIA-F: ^540186^GCGGGCGGTAAGAATTCTTTGCTTG^540210^	CP002005.1	196
	mboIA-R: ^540381^AATGTGGGTAAACATTGATTAAATCGGCAT^540352^		
*mboIB*	mboIB-F: ^541586^ATGAAAAAATTAAATGGTGATAAACAAGCC^541605^	CP002005.1	339
	mboIB-R: ^541924^TTACACCAAACTAATCTGCTCACGA^541900^		
*dpnI*	dpnI-F: ^540572^ GCAAATGAATACAGTAGTAAGGCTC^540596^	CP002005.1	743
	dpnI-R: ^541314^GCATATTTTACCTTTTTATATCGAC^541290^		
*MCR_0360*	t3rme-F: ^376345^ACGCATGACTATCTATTGGTTTATG^376369^	CP002005.1	1075
	t3rme-R: ^377419^AAAATGCTTTGTCCAATACAGTGGT^377395^		
*MCR_0361*	t3rme2-F: ^377989^ACAAACATTGAAATTTTGGTGATGA^378013^	CP002005.1	1080
	t3rme2-R: ^379068^ATAAATTCGCTCGCCTTTTTGATTC^379044^		
*mcbA*	mcbA-F:^1455^CCCTATCCGCCCAAAAAATAGATTG^1479^	NC_011131.1	1022
	mcbA-R: ^2476^ATTTTGTCCTGCATGAGCAGTTTTG^2452^		
*mcbB*	mcbB-F: ^3366^GCTGACAAGGATTTATTAACCACGC^3390^	NC_011131.1	691
	mcbB-R: ^4056^AGGCGATAAATGCCATTAAAATACC^4032^		
*mcbC*	mcbC-F: ^4952^ATGAAATATAAAAAGTTACCCATTGC^4977^	NC_011131.1	306
	mcbC-R: ^5257^TTACCATTTTTTAGTAACTCCAACC^5233^		
*mcbI*	mcbI-F: ^5251^ATGGTAAAGACAATAGGTTTAGCATGGA^5278^	NC_011131.1	225
	mcbI-R: ^5475^TTATTGAGCTGCGCTACTATTTTTGC^5450^		
*rumA*	rumA-F: ^164872^AAACAGTCCGTACTAAAAGAGCTGCTCA^164899^	CP002005.1	396
	rumA-R: ^165267^CAATTTCGCCAAGTCTTCATCATCAAGC^165240^		
*rplF*	rpIF-F: ^1553046^ATGTCTCGTGTGGCTAAAGCCCCAGTAA^1553073^	CP002005.1	507
	rpIF-R: ^1553552^AACAACTTCATCGCTATAACGAACACCC^1553525^		
*MCR_0016*	ATPase-F: ^19342^TTTAAGCAAGTTTCATTTAACTATGGT^19368^	CP002005.1	284
	ATPase-R: ^19625^TTTTCACGCACTGTACGATGTAGTAGC^19599^		
*MCR_0020*	spoU-F: ^24189^AGCACCGATAACACCGACTGTGGCTAA^24215^	CP002005.1	230
	spoU-R: ^24418^GAGTTAATCGACGCATGCCATCACCTT^24392^		
*MCR_1465*	mtrF-F: ^1464808^TTGGGGAATTTATTGCCCCATCCTG^1464832^	CP002005.1	793
	mtrF-R: ^1465600^TCAGCATACTTACCCCCGCCCAAAC^1465576^		
*copB*	copB-F: ^512291^CAGCGTGAAACCTACCAAAAGTTAACC^512317^	CP002005.1	671
	copB-R: ^512961^TTAAAACCAATCTCGGTATTGCGTGCT^512935^		
*rrmA*	rrmA-F: ^1646694^GAAAACCTACCAATGTGCCAATCAA^1646718^	CP002005.1	455
	rrmA-R: ^1647148^CATCACAAAGCACTCGTCGTATTTC^1647124^		

**Table 4:** The mutations of A2144T, A2330T and C2480T of 23S rRNA in four operons of the four *M. catarrhalis* isolates used in this study.

23S rRNA_position (NR_103214.1)	Single-base mutation	In the genome position (CP002005.1)	11XR4410	13R13685	11XR1696	13R13726
*A2144*	A− > T	319857	+	−	−	−
		1441446	+	−	−	−
		1680011	+	−	−	−
		1824585	+	−	−	−
*A2330*	A− > T	320043	+	+	−	−
		1441260	+	+	−	−
		1679825	+	+	−	−
		1824399	+	+	−	−
*C2480*	C− > T	320193	+	+	−	−
		1441110	+	+	−	−
		1679675	+	+	−	−
		1824249	+	+	−	−

**Table 5:** Evaluation of SNPs in clinical isolates.

Genes_SNPs	Single-base mutation	Verification in clinical isolates (Resistant isolates [R], n = 21; Susceptible isolates [S], n = 52)	*P* value
*rumA_619*	C− > T	R: 10/21 S: 2/52	<0.0001
*rplF_140*	T− > C	R: 8/21 S: 1/52	<0.0001
*MCR_0020_643*	G− > A	R: 8/21 S: 0/52	<0.0001
*MCR_1465_270*	T− > G	R: 4/21 S: 0/52	0.0055
*copB_1348*	C− > A	R: 9/21 S: 8/52	0.0292
*rrmA_238*	G− > A	R: 6/21 S: 2/52	0.0058
*23S rRNA_2330*	A− > T	R: 21/21 S: 0/52	<0.0001
*23S rRNA_2480*	C− > T	R: 13/21 S: 0/52	<0.0001
*23S rRNA_2144*	A− > T	R: 3/21 S: 0/52	0.0214
*MCR_0016_1249*	A− > G	R: 3/21 S: 2/52	0.1395
*MCR_1465_695*	G− > A	R: 13/21 S: 31/52	1.0000
*copB_1205*	A− > C	R: 15/21 S: 23/52	0.0680
