# Supplementary material for: Use of next generation sequence to investigate potential novel macrolide resistance mechanisms in a population of Moraxella catarrhalis isolates
Source: Sci Rep. 2016 Oct 24;6:35711. doi: 10.1038/srep35711 (PMC5075928; doi:10.1038/srep35711)
Supplement: Supplementary Information [file srep35711-s1.doc]

**Use of next generation sequence based comparative genomics to investigate potential novel macrolide resistance mechanisms in a population of *Moraxella catarrhalis* isolates.**

Ya-Li Liu^1†^, Dong-Fang Li^2,3,4†^, He-Ping Xu^5†^, Meng Xiao^1^, Jing-Wei Cheng^1^, Li Zhang^1^, Zhi-Peng Xu^1^, Xin-Xin Chen^1^, Ge Zhang^1^, Timothy Kudinha^6,7^, Fanrong Kong^7^, Yan-Ping Gong^3,4^, Xin-Ying Wang^3,4^, Yin-Xin Zhang^3,4^, Hong-Long Wu^2,3,4,*^ , Ying-Chun Xu^1,*^

^1^Department of Clinical Laboratory, Peking Union Medical College Hospital, Peking Union Medical College, Chinese Academy of Medical Sciences, Beijing 100736, China;

^2^Wuhan National Laboratory for Optoelectronics, Huazhong University of Science and Technology, Wuhan, Hubei 430074, China;

^3^Binhai Genomics Institute, BGI-Tianjin, BGI-Shenzhen, Tianjin 300308, China;

^4^Tianjin Translational Genomics Center, BGI-Tianjin, BGI-Shenzhen, Tianjin 300308, China;

^5^Department of Clinical Laboratory, the First Affiliated Hospital of Xiamen University, Xiamen, China;

^6^ Charles Strut University, Leeds Parade, Orange, New South Wales 2687, Australia;

^7^Centre for Infectious Diseases and Microbiology Laboratory Services, ICPMR – Pathology West, Westmead Hospital, University of Sydney, Darcy Road, Westmead, New South Wales 2145, Australia.

†Ya-li Liu, Dong-fang Li, and He-ping Xu contributed equally to this work.

^*^Corresponding authors:

Professor Ying-Chun Xu

Department of Clinical Laboratory, Peking Union Medical College Hospital, Peking Union Medical College, Chinese Academy of Medical Sciences, Beijing 100730, China.

Phone: 86-10-6915-9766;

Fax: 86-10-6915-9766;

E-mail: xycpumch@139.com.

Professor Honglong Wu

Wuhan National Laboratory for Optoelectronics, Huazhong University of Science and Technology, Wuhan, Hubei, 430074, China;

Binhai Genomics Institute, BGI-Tianjin, BGI-Shenzhen, Tianjin, 300308, China;

Tianjin Translational Genomics Center, BGI-Tianjin, BGI-Shenzhen, Tianjin, 300308, China

**Supplementary Table S1. Annotations of the 12 selected differential genes and 12 selected informative candidate SNPs**

| **Gene name** | **Annotation** | **References** |
| --- | --- | --- |
| *MCR_1794* | This gene encodes a kind of Type II restriction endonuclease (REase), which is a sequence-specific endonuclease that recognizes short DNA sequences and cuts the DNA at defined positions within or close to the recognition sequence. | 22, 23, 24 |
| *MCR_1795* | This gene encodes a kind of DNA -cytosine methyltransferase enzyme (DCMTase). This enzyme catalyzes the methyl transfer from donor co-factor *S*-adenosylmethionine to the target cytosine. A bacterial DCMTase not only extrudes the substrate cytosine, but also induces frame-shifted base pairing and the formation of a large gap in the duplex DNA recognition site. | 25, 26, 27, 28 |
| *lgt2B/C* | A 765 bp long gene encoding a putative β-1,4-galactosyltransferase, is found in all three serotypes of *M. catarrhalis* as two alleles: lgt2A is only found in serotype A strains, while *lgt2B/C* is shared by serotypes B and C strains. The two alleles correlate with structural differences, as serotypes B and C strains have galactose added to terminal a-D-Glc and/or a-D-GlcNAc residues of the 1→6 and 1→4 substituted chains, whereas serotype A strains have galactose addition to the terminal a(1→2) Glc of the 1→6 chain. | 29, 30, 31,32,33 |
| *mboIA* | Modification methylase MboIA | None |
| *mboIB* | Modification methylase MboIB | None |
| *dpnI* | This gene encodes a restriction endonuclease (REase), which only cuts the methylated sequence G^m^ATC when the adenine residue is methylated. | 34, 35, 36, 37 |
| *MCR_0360* | Type III restriction-modification system restriction endonuclease | None |
| *MCR_0361* | Type III restriction-modification system restriction endonuclease | None |
| *mcbA*, *mcbB*, *mcbC*, *mcbI* | The *mcbA*, *mcbB*, *mcbC*, and *mcbI* (four-gene cluster) were first detected in *M. catarrhalis* strain E22 plasmid pLQ510. DNA and Reverse transcriptase-PCR all showed that the *mcbA*, *mcbB*, *mcbC*, and *mcbI* ORFs were linked. The two genes *mcbA* and *mcbB* in pLQ510 encoded proteins predicted to be involved in the secretion of a bacteriocin. *mcbC* encoded a protein which had some homology to double-glycine bacteriocins produced by other bacteria. The *mcbI* immediately downstream from *mcbC* encoded the cognate immunity factor. | 38 |
| *rumA* | 23S rRNA (uracil-5-)-methyltransferase RumA | None |
| *rplF* | 50S ribosomal protein L6 | None |
| *MCR_0016* | Multidrug efflux pump ABC transporter ATPase subunit | None |
| *MCR_0020* | This gene encodes a SpoU family rRNA methyltransferase. In ribosomal RNA (rRNA), methylation may act as a checkpoint in ribosome subunit assembly, influence the process of translation, and alter resistance to certain antibiotics. RNA MTases have been identified as clinically significant resistance determinants to a number of ?antibiotics, including the aminoglycoside (Arm MTase) and erythromycin (Erm MTase) antibiotics. | 39, 40, 41, 42, 43, 44, 45 |
| *MCR_1465* | Putative efflux pump component MtrF | None |
| *copB* | Outer membrane protein CopB | None |
| *rrmA* | This gene encodes 23S ribosomal RNA G745 methyltransferase, which methylates the N1 position of nucleotide G745 in hairpin 35 of *Escherichia coli* 23S ribosomal RNA (rRNA). RrmA resembles the homologous methyltransferase TlrB (specific for nucleotide G748) as well as the Erm methyltransferases (nucleotide A2058), in which all these enzymes methylate their target nucleotides. Methylation of nucleotide G748 and A2058 of 23 S rRNA confers tylosin resistance, suggesting that these rRNA regions are adjacent and are part of the binding site for MLS_B_ drugs. | 46, 47 |
